# Review of how we should define (and measure) adherence in studies examining older adults' participation in exercise classes

**DOI:** 10.1136/bmjopen-2016-011560

**Published:** 2016-06-23

**Authors:** H Hawley-Hague, M Horne, D A Skelton, C Todd

**Affiliations:** 1 School of Health Sciences, The University of Manchester and Manchester Academic Health Sciences Centre, Manchester, UK; 2 School of Nursing, University of Bradford, Bradford, UK; 3 School of Health, Glasgow Caledonian University, Glasgow, UK

**Keywords:** PREVENTIVE MEDICINE, REHABILITATION MEDICINE, STATISTICS & RESEARCH METHODS, SPORTS MEDICINE, GERIATRIC MEDICINE

## Abstract

Exercise classes provide a range of benefits to older adults, reducing risk of illness, promoting functional ability and improving well-being. However, to be effective and achieve long-term outcomes, exercise needs to be maintained. Adherence is poor and reporting of adherence differs considerably between studies.

**Objective:**

To explore how adherence to exercise classes for older people is defined in the literature and devise a definition for pooling data on adherence in future studies.

**Design:**

Methodological review of the approaches used to measure adherence.

**Methods:**

A review of the literature was carried out using narrative synthesis, based on systematic searches of MEDLINE, EMBASE, CINAHL and PsychINFO. 2 investigators identified eligible studies and extracted data independently.

**Results:**

37 papers including 34 studies were identified. 7 papers (7 studies) defined adherence as completion (retention). 30 papers (27 studies) identified adherence using attendance records. 12 papers (11 studies) based adherence on duration of exercise and 5 papers (4 studies) specified the intensity with which participants should exercise. Several studies used multiple methods.

**Conclusions:**

There was little consensus between studies on how adherence should be defined, and even when studies used the same conceptual measure, they measured the concept using different approaches and/or had different cut-off points. Adherence related to health outcomes requires multiple measurements, for example, attendance, duration and intensity. It is important that future studies consider the outcome of the intervention when considering their definition of adherence, and we recommend a series of definitions for future use.

**Strengths and limitations of this study**

- The way that older adults' adherence to community exercise classes is reported differs considerably between studies.
- Data cannot be pooled for meta-analysis.
- We define how adherence should be measured dependent on the outcomes of the intervention.

## Introduction

Promoting exercise among the older population is an important public health and clinical issue.^1^ ^2^ Exercise reduces illness, improves functional ability and improves well-being.^3^ However, to achieve long-term benefits, older adults have to continue to do exercises and maintain activity either in exercise classes or alone (ie, they have to adhere to gain benefit). Continuation of exercises by older adults in the general population and within a rehabilitation setting is poor, which leads to little gain or even deterioration of function.^4–6^

There is a broad range of definitions of adherence used in the literature. In the general exercise literature, adherence is defined as successful if participants complete a prescribed exercise routine for at least two-thirds of the time.^7^ This definition is very much related to functional improvements, as consistent exercise is needed to see improvements in, for example, strength and balance.^8^ It not only bases adherence on the number of sessions and their intensity, but also provides a cut-off point (two-thirds or more of the prescribed sessions is adherent). Self-report methods of exercise performance in terms of minutes or hours of exercise carried out, using measures such as the Community Healthy Activities Model Program for Seniors (CHAMPS) physical activity questionnaire, have also been used.^9^ Recent research looking at exercise classes considered two different measures of exercise continuation: class adherence, which was defined as still attending at follow-up,^10^ and class attendance (number of classes attended over a set period). Results indicate there is a difference between attendance and adherence (as defined in the study), since some variables measured only relate to one concept, indicating that different concepts are being measured.^10^ This raises questions with regard to the way that adherence is defined and the cut-off points used as part of that definition. Attendance could be seen as a subset of adherence and continues to be an important measure in its own right. For this study, we focus on the broader concept of adherence, as the outcome and impact of an intervention could be different depending on the definition and measurement used.

The definition of adherence becomes particularly interesting when applied to exercise classes, as there is less reliance on self-report data. There seems to be no agreed definition of adherence in relation to exercise classes. This could have important implications for general community-based and rehabilitation exercise classes. Therefore, a review of the literature has been carried out (based on systematic searches), to explore definitions of adherence to exercise classes for older adults. Visek *et al*^11^ discuss four measures used for adherence to structured exercise in trials: (1) completion (ie, retention), (2) attendance (the number of sessions attended over the follow-up period), (3) duration adherence (how long they exercise for at each session) and (4) intensity adherence (the physical exertion). These measures will provide the framework for the review, with additional measures added if identified. This review explores how adherence to exercise classes is defined in the literature and makes suggestions for a consistent definition for future studies, so as to guide study design and so that meta-analyses of adherence to group exercise interventions can be performed in the future.

## Methods

### Search strategy and selection criteria

We searched the Cochrane library, and then we undertook systematic searches of MEDLINE, EMBASE, CINAHL and PsychINFO. No date restrictions were placed on the search and all relevant evidence was included if in the English language. A direct journal search was also carried out on *Age and Ageing* and *Journal of Aging and Physical Activity*. Search terms were both free-text and MeSH headings and were combined with Boolean operators. Key search terms included ‘older adults’, ‘seniors’, ‘exercise’, ‘strength’ and ‘balance’ and ‘adherence’, ‘maintenance’ and ‘compliance’. The terms strength and balance were included as additional terms as community classes for falls prevention are often referred to as strength and balance training/classes rather than exercise. The electronic searches were carried out up to 1 June 2015. The searches were originally carried out for a systematic review on uptake and adherence to exercise classes and have been adopted for this review. Two investigators identified eligible studies and extracted data independently, where there was any disagreement a decision was made through discussion with a third investigator.

### Types of study

All types of quantitative study designs were included. Most studies in this area of research are exploratory and there are few randomised controlled trials.

### Inclusion/exclusion criteria

#### Participants

We include all quantitative studies including older adults aged 50 and above. As pre-retirement age adults often have different needs,^12^ the study participant's mean age had to be ≥60 years.

#### Types of interventions

We focus on community-based exercise classes or strength and balance classes. This includes community-based exercise classes in trials. The classes had to have more than one fitness component, as the evidence indicates this is required to prevent/manage many conditions.^2^ ^13^ These components were defined as including aerobic, strength, balance, stretching and mobility. Studies considering Pilates and Tai Chi were excluded in the original review and so are not included here either. There is no agreed definition of an exercise class. We combine the standard definition for exercise^14^ with the concept of a directed class to define the exercise classes included in this review as ‘a group of people gathered together to follow a leader or instructor to carry out planned, structured and repetitive bodily movement done to improve more than one component of physical fitness’.

To be included, studies had to report adherence (however that was defined) to an exercise class, but adherence did not have to be the primary outcome measure. Narrative synthesis was adopted.

## Results

**Figure 1:**
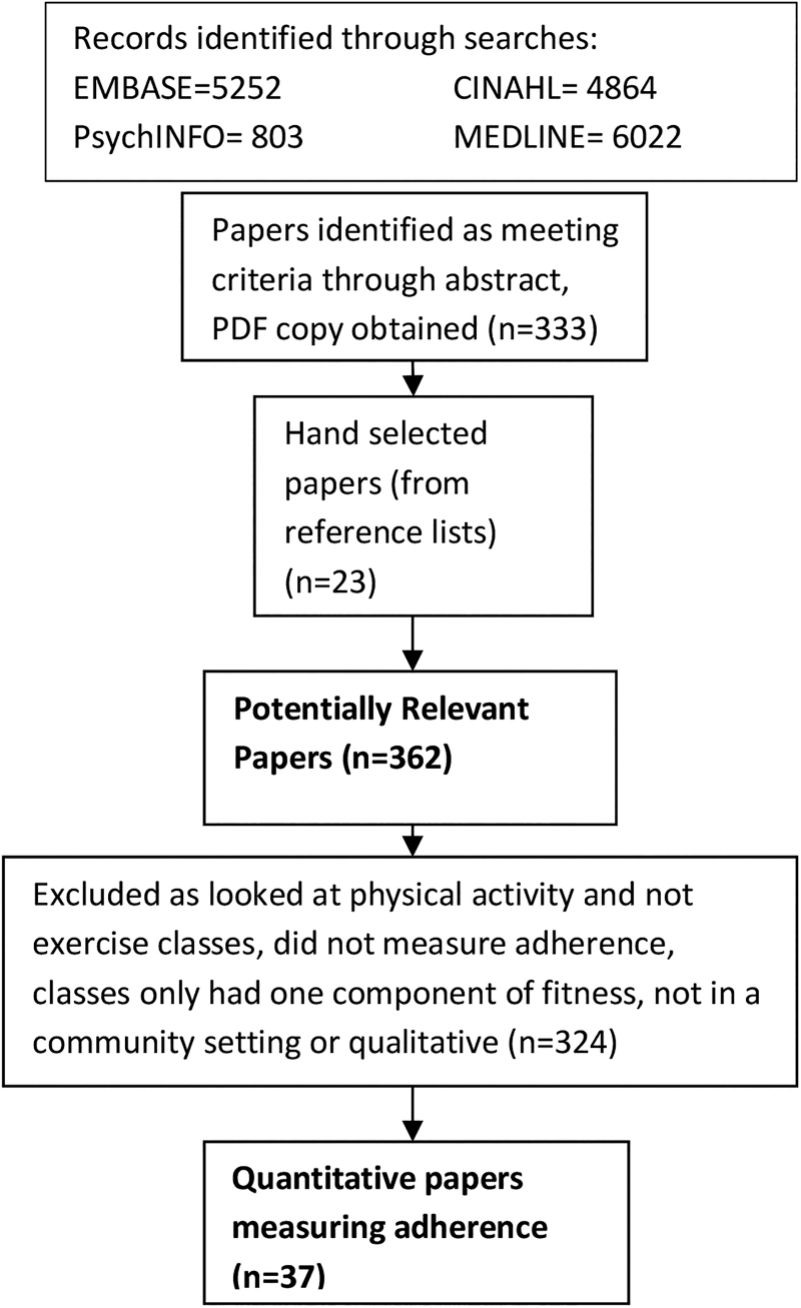
PRISMA diagram.

Figure 1 presents the PRISMA diagram for our review process. The searches were originally carried out for a separate systematic review, but for this review, we excluded papers that did not measure adherence (the original review also looked at uptake) or because they were qualitative. Online supplementary table S1 presents details of the 37 papers (34 studies) identified which fulfilled inclusion criteria for this review. Below we identify the different ways these papers measured adherence, and the implications of defining and measuring adherence in that way.

10.1136/bmjopen-2016-011560.supp1 Supplementary table Details of studies included in the review.

### Completion (ie, retention)

Seven papers (seven studies) defined adherence as completion or conversely lack of adherence as drop-out.^10^ ^15–20^ Sometimes this was also assessed alongside another measure such as percentage of or number of attendances.^10^ ^16^ ^21^ In one study, completion (adherence) was whether participants returned after a 10-week break^15^ and another study described adherence as actual full completion of the programme and present at the last class.^19^

Drop-out was described in different ways in the studies. It was described as withdrawal from a programme/not returning to the class^10^ ^20^ ^21^ or withdrawal due to health reasons after missing a number of sessions. Time until drop-out^18^ was also measured in one study, which was the number of days between first and last attended class.

### Attendance

Thirty papers (27 studies) defined adherence by using attendance records.^9^ ^15^ ^16^ ^20–46^ One paper (one study) measured attendance but described retention as adherence.^10^ Fourteen papers (11 studies) defined adherence as the percentage of classes attended.^9^ ^20–23^ ^25^ ^29–31^ ^35^ ^39^ ^42–44^ Authors calculated percentage in a number of ways. In Estabrooks and Carron^16^ attendance was calculated as a percentage of total number of classes available over the 4-week period. Ecclestone and Paterson^21^ calculated the percentage of classes attended out of the actual number of sessions offered for each class in each calendar month. Hays *et al*^33^ calculated the mean number of classes attended, but also used exercise intensity as a measure. Eight papers (eight) studies defined different ordinal levels of adherence based on percentage of attendance thresholds, classified in different ways.^24^ ^34^ ^36–38^ ^40^ ^41^ ^44^ For example, in Stineman *et al*,^38^ high adherence was classed as attending all sessions, whilst Sjösten *et al*^24^ defined high adherence as 66.7–100% attendance and Grove and Spier^36^ defined high adherence as the percentage of older adults who attended 90–100% of sessions. In other papers,^40^ ^41^ ^44^ high attendance was defined as participation in >75% of all exercise sessions. Some papers set a minimum attendance for low adherers, such as <30% of exercise classes^37^ or <15 out of 20 sessions.^34^ Mills *et al*^28^ called ‘maintained participation’, attending at least one class a month, which was assessed through self-report, but validated by attendance records. Keogh *et al*^45^ described high attendance as having attended one session a week over the previous 3 months. Finally, one paper also included drop-out as well as attendance in a combined adherence measure, for example, Estabrooks and Carron^15^ based adherence on attendance over 6 weeks (percentage of classes attended) but also return rate after a 10-week break.

### Duration adherence

Twelve papers (11 studies) based adherence on duration of exercise, which was measured in a variety of different ways.^9^ ^29^ ^30^ ^39^ ^42^ ^43^ ^47–52^ Duration adherence was often used to measure self-reported exercise that included exercise carried out within the classes and outside the classes. This was primarily used for longitudinal follow-up after a time-limited intervention.^30^ Two papers (two studies) used self-report exercise and calculated a level of physical activity using, for example, the physical activity questionnaires, PACE^29^ ^30^ or the Yale Physical Activity Survey (YPAS).^39^ One paper (one study) just asked participants to record whether they had exercised 2–3 times a week over the set time period using a Likert scale.^47^ Three papers (three studies) asked participants to record the number of minutes they were physically active,^49–51^ whereas five papers (four studies) asked participants to record adherence to predefined minutes, for example, 30 min, three times a week.^9^ ^42^ ^43^ ^48^ ^52^

### Intensity adherence

Five papers (four studies) specified the intensity with which participants should exercise.^33^ ^42^ ^43^ ^48^ ^52^ Hays *et al*^33^ stated adherence as a minimum of 20 min of continuous exercise at 55–70% of maximum heart rate (moderate intensity as defined by the American College of Sports Medicine, ACSM). Litt *et al*^52^ asked that participants exercise at ‘moderate intensity’ as per the prescribed exercise regime. Caserta and Gillett^48^ and Gillett *et al*^42^ ^43^ asked participants to report how many times they exercised three times a week for 30 min at 60–80% of maximum heart rate.

### Lack of uptake

Ecclestone and Paterson^21^ looked at attendance to a range of programmes and defined lack of adherence as not registered on any programmes, not attending a single session over a 12-month period or not returning to a class within the 12-month tracking period. Two of these three measures should be described as lack of uptake, rather than adherence.

## Discussion

There is clearly confusion in the literature about the definition of adherence, and even in differentiating adherence from uptake. There is very little consensus in the papers reviewed on how adherence should be defined, and even when studies used the same conceptual approach, measurement used different approaches and/or had different cut-off points for what counted as being adherent.^53^ The majority of papers/studies included in this review focused on attendance of classes, particularly percentage of attendance as the measure of adherence. Very few studies looked at exercise intensity and this was only used alongside another measure.^33^ ^42^ ^43^ ^48^ ^52^

Clearly adherence can be defined and measured in a variety of ways. How it is done should depend on the purpose of measurement. If adherence is being measured for management purposes, so as to ascertain if a programme is viable in a community, measurement in terms of weeks attended may suffice. This measurement will inform whether the class can continue to be provided and is economically viable, since regular weekly attendance is important, as if large numbers of participants are away from classes for long periods of time the class may become unviable. If, however, adherence is being measured in a study which is looking to see if the intervention brings about a health gain, for example, for maintenance of strength and balance and to reduce falls risk, then the definition of adherence needs to focus on a number of measurements. Using falls prevention as an example, the definition should be based on the evidence base for falls prevention, and thus completion (ie, retention), attendance, duration and intensity adherence are all important to indicate whether older adults receive adequate dose of strength and balance training on an ongoing basis to prevent falls.^13^

If adherence is being measured for motivational reasons or even to test whether the group is cohesive, then we may want to focus on measuring attendance and completion (retention). Completion (retention) when used as a measure alone may mean that an individual may have missed a substantial number of classes, but could still be called adherent. Attendance when used as a single measure may indicate a lack of commitment, when the individual's attendance has been affected by ill health or vacation and they are committed enough to always return to the class.^54^ Completion and attendance as a combined measure helps us to understand participants' attitudes towards and commitment to the class, as well as their satisfaction with the class in terms of physical and social outcomes. They may not be attending for valid and practical reasons (ill health, caring duties, long holidays), and this combined measure may better reflect real life.

For research purposes, adherence needs to reflect the outcomes that are being measured and there needs to be a consensus agreement on which measures are used for which outcomes. The way that each type of adherence is measured also varies and this causes issues for data pooling, meta-analysis and comparison of interventions. It is suggested that a consensus agreement is reached on when different types of measurement of adherence are used to provide consistency in the literature. In the absence of an agreed consensus, we recommend the following clear definitions be used.

1. Health outcomes: completion (ie, retention), attendance, duration and intensity adherence.
2. Group cohesion/motivation: completion (ie, retention) and attendance.
3. Financial viability: attendance.

The cut-off points for indicating each concept also differ, and therefore we also recommend how each definition is measured (based on those used most frequently within the literature and our suggestion of when different definitions should be used):

1. Completion (retention): those who are still attending the class/still attending at follow-up. Non-completion includes withdrawal from the class or where there is no formal withdrawal measured as not attending at follow-up (without reason given to the instructor).
2. Attendance: percentage of classes attended out of the actual number of sessions offered.
3. Duration: adherence to predefined minutes, for example, 30 min, three times a week.
4. Intensity: ‘moderate intensity’ as per the prescribed exercise regime. Moderate intensity may differ dependent on the type of programme (eg, strength and balance or aerobic), but the ACSM guidelines should be taken into consideration.

Even if these definitions of the types of adherence gain consensual acceptance by the research community, the measurement of adherence is not always valid or reliable. Minutes of exercise as a measure, for example, may be unreliable as this measure is often self-reported and there are a number of problems with self-report data.^55^ There is potential to use technology to calculate number of minutes of exercise, types and intensity of exercise. The use of sensors could enable us to accurately measure older adults exercise within ‘real time’ and work has been carried out exploring the accurate recording of movement.^56^ While use of sensors could help solve the problem of measuring adherence, they might of themselves provide a new source for a Hawthorne effect.

The limitations of this study are that it only provides definitions of adherence for exercise classes and not general physical activity. We believe that the definition of adherence for physical activity will differ because there is an increased reliance on self-report data. We also excluded studies which looked at Tai Chi and Pilates. This was because there are sufficient studies on these types of exercise class for a separate review. Both Tai Chi and Pilates have the potential to provide important benefits to older people, and therefore further research is required to assess whether our recommended definitions can also be applied to these interventions.

Although in this study we only define adherence to exercise classes, some of the included studies looked at changes during and after the exercise class and therefore use a self-reported exercise duration measure throughout their studies that is not always directly related to the time spent exercising in the class. If we had excluded studies which carried out follow-up after the class, then this may have allowed us to present simpler results. However, their inclusion highlights an important complexity that has arisen in the literature that needs to be considered and our definition take into account these different measurements used.

Our cut-off point for intensity of exercise focuses on moderate-intensity exercise. We know that even low-intensity exercise has benefits for older adults.^57^ However, all of the included studies that measured exercise intensity based their measure of adherence on moderate intensity. Our proposed cut-off point takes into consideration that moderate-intensity exercise may differ dependent on the individual and type of programme.

It is important that future studies consider the outcome of the intervention when considering their definition of adherence but also that the way this is measured is clearly outlined so as to enable comparison and provide a full picture.
