# Supplementary material for: Review of how we should define (and measure) adherence in studies examining older adults' participation in exercise classes
Source: BMJ Open. 2016 Jun 23;6(6):e011560. doi: 10.1136/bmjopen-2016-011560 (PMC4932302; doi:10.1136/bmjopen-2016-011560)
Supplement: Supplementary table — Details of studies included in the review. [file bmjopen-2016-011560supp_table.pdf]

Table 1: Details of studies included in the review.

Author, Date, Location	Main study aim	Study Design	Sample	Intervention	Adherence
Liu et al, 2014 U.S. [46]	To determine if sarcopenia modulates the response to a physical activity intervention in functionally limited older adults.	Randomised Controlled Trial	N= 177 (mean age 77.0) 71.1% female, 81.3% white Caucasian	**Intervention:** aerobic, strength, balance and flexibility exercises for 12 to 18 months, During weeks 1 to 8, 3 x weekly sessions were supervised at the field centre. From weeks 9 to 24, supervised sessions were 2x weekly and home-based exercises initiated. At 24 weeks, subjects transitioned to a home-based program with an optional weekly supervised session.	**Measure:** Number of sessions attended was compared to number of sessions available, excluding closings. For comparison of groups, the total number of sessions for each group at each site for the study was used.
Freiberger et al, 2013 Germany [44]	Feasibility of reaching functionally declined, but still	Randomised Controlled Trial	N=378 (mean age= 78.1), 75.4% female. No	**Intervention:** 16-week intervention included progressive and challenging balance,	**Measure:** participated in more than 75% of the supervised group sessions.

	independent older persons at risk of falls through their general practitioner (GP) and reduce their physiological and psychological fall risk factors with a complex exercise intervention		ethnicity stated community dwelling	gait, and strength exercise as well as changes to behavioural aspects. Sixteen sessions, once per week for 60 minutes, were supervised, and the participants added at least one unsupervised session starting from week 5.	Trained according to the protocol while unsupervised.
Courneya et al, 2012. Canada [49].	Examine the predictors of exercise adherence in the Alberta Physical Activity and Breast Cancer Prevention (ALPHA) Trial	Randomised Controlled Trial	N=160 No mean age stated (aged 50-74 with more participants >60) 100% women Ethnicity not stated community dwelling.	**Intervention:** Participants asked to perform at least 3 sessions/wk (approximately 123 of the 200 minutes) in supervised exercise at a fitness facility and up to 2sessions/wk (77 minutes) in unsupervised exercise.<br><br>Specific interventions to improve adherence: Individualised exercise programme with regularly scheduled sessions, automatic telephone follow-up, plans for sessions missed because of	**Measure:** exercise adherence was weekly minutes of total, supervised, and unsupervised exercise excluding warm-up and cool-down periods. Supervised exercise minutes measured objectively by exercise trainers. Unsupervised exercise minutes assessed by exercise logs completed on weekly basis. Total exercise was sum of the supervised and unsupervised exercise minutes.

				vacations or illness, comprehensive educational package, group sessions, positive social interaction, donated incentives awarded at different milestones, regular newsletters, and study website.	
Evers et al, 2012. Germany [22].	Whether social cognitive variables and coping plans predict adherence to physical and mental activity intervention.	Randomised Controlled Trial	N=171 (mean age=73.7) 100% women, no ethnicity stated, community dwelling.	**Intervention:** **Physical exercise-** 3 times a week 90 minute multi-component sessions for 26 weeks. **Computer course-** sessions for 26 weeks.	**Measure:** defined as the number of course units attended, was recorded by all trainers for each participant in each course unit (percentage attended).
Evers et al, 2012 Germany [23].	Whether telephone or self-administered coping strategies affect long-term adherence.		N=86 (as above)	**Intervention:** As above. After 6 weeks (18 sessions) participants either had telephone-assisted (n=43) or a self-administered (n=43; control group) coping planning intervention.	**Measure:** As above.
Stineman et al, 2011. U.S [38].	To assess the recruitment, adherence, and	Randomised Controlled Trial	N=204 (mean age=76)	**Intervention:** endurance, resistance, and balance training.	**Measure:** Proportion of on-site exercise sessions attended out of a potential of 7 sessions in total.

	retention of urban elderly, pre-dominantly African Americans to a falls reduction exercise programme.		74.5% women, 88.7% African American, community dwelling.	Once a week class for first month, then monthly class and home exercise, with monthly home visits. 4 month programme. **Control:** generic pamphlet that discussed the benefits of walking three times a week for 30 min a day.	High adherence defined as attendance at all 7 classes. Home exercise: number of weeks exercised 3 or more days that week (self-report), percentage of weeks. High adherence defined as exercising at least 3 days every week for the 12 weeks.
Phillips et al, 2010. U.S [17].	Describe the characteristics of physical activity participants undergoing medical suspensions and distinguish those who never returned to the physical activity intervention from those who successfully returned to complete the intervention.	Randomised Controlled Trial	N=213 (mean age=76.5) 68.5% women, 75.1% white Caucasian. healthy sedentary community dwelling	**Intervention:** Group exercise 3 times per week, reduced to once per week after 8 weeks until 12 months. Intervention delivered by: instructor **Control:** Successful ageing education.	**Measure:** Participants classified as medically suspended if missed three or more consecutive sessions of centre-based physical activity (adoption and transition stage) or 2 or more weeks of home-based physical activity (maintenance) because of a health event
Fielding et al, 2007. U.S [9]	Whether older adults can reasonably participate and	Randomised Controlled Trial	N=213 (mean age=76.5) 68.5% women	Group exercise 3 times per week, reduced to 2 times a week after 8 weeks and then reduced	**Measure:** CHAMPS physical activity questionnaire. Above 150 minutes a week (self-report).

	adhere to a regular programme of physical activity.<br><br>Looked at physical functioning but related to self-report adherence measure.		24.9% non-white. healthy sedentary community dwelling	to 1 a week at 25 weeks.	Attendance at sessions: percentage of available sessions (excluding sessions missed because of medical suspension).<br><br>Home activity logs<br><br>Adherence measured including intention to treat.
Sjosten et al 2007. Finland [24]	Determine the adherence rates and the predictors of adherence in four key activities of a multifactorial fall prevention trial	Randomised Controlled Trial	N= 293 (mean age=73) 86% female no ethnicity stated Community dwelling fallers	Multi-factorial falls prevention programme including multi-component class twice a month for 12 months.	**Measure:** Determined as a participation rate (number of attendances from number offered) in group session.<br>(i) 0% adherence rate (non-adherence), (ii) 0.1–33.3% adherence rate (low adherence), (iii) 33.4–66.6% adherence rate (moderate adherence) and (iv) 66.7–100% adherence rate (high or full adherence).
McAuley, et al, 2003. U.S [30] & McAuley, et al, 2003. U.S [31]	Examined predictors of long-term exercise behaviour in older adults following a 6-month randomised controlled exercise trial	Randomised Controlled Trial	N= 89 (mean age=66.0) 94% white Caucasian healthy sedentary community	**Intervention:** Walking **Control:** exercise class 3 times a week for 1 hour, 6 months.	**Measure:** During trial attendance records were collected.<br><br>However analysis related to Physical Activity Scale for the Elderly looking at physical activity levels at 6- and 18-month follow-up (self-report).
Gillett, White & Caserta	To test the effect of two nurse-delivered exercise/education	Randomised Controlled Trial	N=76 exercise group (mean	**Intervention:** exercise/education programme 3 times a	**Measure:** Self-report but also includes attendance records of class for first 16 weeks.

1996 U.S [43]	programmes specifically designed for obese, older women		age=64.7). 98% white Caucasian sedentary, overweight community dwelling.	week, 16 weeks Intervention delivered by: Nurse.	Analysis included self-report activity.
Gillett &Caserta 1996 U.S [42]	To test the effect of two nurse-delivered exercise/education programmes specifically designed for obese, older women.	Randomised Controlled Trial	N=76 exercise group (mean age=64.7). 100% women 98% white Caucasian sedentary, overweight community dwelling.	**Intervention:** exercise/education programme 3 times a week, 16 weeks Intervention delivered by: Nurse. **Intervention 2:** Health education sessions where encouraged to exercise 3 times a week at home, 16 weeks. **Control:** Test taken but no further intervention.	**Measure:** Attendance records, percentage of classes attended. Also, self-report particularly after 16 weeks. Self-report diaries. 3 times a week for 30 minutes at 60-80% of maximal heart rate reserve (MHRR), although this is not clear in the paper. Analysis tended to be based on self-report.
Hughes, Seymour et al, 2006. U.S [51]	Present final outcomes of multi component Fit and Strong! Intervention.	Randomised Controlled Trial	N=115 (mean age=73.3) 80.6% women. 69.4% white Caucasian mild to moderate osteoarthritis. community dwelling	**Intervention:** multi-component class and behaviour change 90 minutes, 3 times a week, 8 weeks. Intervention delivered by: Physical Therapists. **Control:** Waiting list.	**Measure:** Monitored attendance at sessions but they were asked to log activity. Frequency (number of times a week) and duration (minutes). Analysis was mostly done with self-report data.
Keogh et al, 2014	To quantify the objective benefits,	Exploratory Intervention	Study 1 N= 62	**Intervention:** group-based resistance,	**Measure:** current attendees defined as averaging at least one

New Zealand [45]	participant perceptions and retention rates of a New Zealand community-based exercise programme for adults (60 years or older).	Study (pre/post and cross sectional study) 3 studies	(mean age=71) 69% women<br><br>Study 2 N= 153 (mean age=72) 63.4% women<br><br>Study 3 N=264 (mean=72) 65% women<br><br>ethnicity not stated for any studies. All community dwelling.	balance, cardiovascular and flexibility training activities, 60 minutes in duration and undertaken twice weekly for 12 weeks.	class a week over the previous three months. Based on attendance records, the length of programme participation (to the closest month) was also determined.
Toto et al, 2012. U.S. [39]	Evaluate effect of participation in multicomponent best-practice exercise and physical activity program (FSAH) on physical activity, ADL performance, physical performance,	Exploratory Intervention Study (pre/post)	N=15 (mean age=78.1) 100% women 100% white Caucasian sedentary, community-dwelling. low-income households	**Intervention:** 10-week intervention included multi-component group exercise sessions and a home exercise program. Group sessions also included key strategies for increasing self-efficacy. Participants met for 60-min group sessions 2 times week.	**Measure:** Yale Physical Activity Survey (YPAS), measuring self-report physical activity.<br><br>Attendance at group sessions, percentage of sessions attended.

	depression.				
McAuley et al, 2011. U.S [31]	To examine the hypothesis that self-efficacy mediates the relationship between self-regulatory processes, such as executive function, and sustained exercise behaviour.	Exploratory Intervention Study (pre/post)	N=177 (mean age=66.4) 65.5% women 91% white Caucasian, community dwelling.	**Intervention:** participants randomised into a walking group or flexibility, toning, and balance (FTB) group. Classes met 3 days per week for approximately 1 hour over 12-month period	**Measure:** Adherence reflects percentage of attendance to exercise classes over the last 11 months of the program. Attendance data were recorded each day by staff, aggregated, and divided by the total possible number of sessions to arrive at percentage attendance.<br><br>n.b attendance was no different for the walking or exercise group.
Sullivan-Marx et al, 2011. U.S [19].	Examined employment of specific recruitment and retention strategies in study evaluating outcomes of moderate activity exercise programme for older African American women	Exploratory Intervention Study (pre/post)	N=52 (mean age=79) 100% women 100% African American community dwelling.	**Intervention:** Three 5-min walking intervals interspersed with two strength and balance intervals. Programme held 5 days/week for 16 weeks; sessions lasted 30 min to 50-min sessions.	**Measure:** Completion of the exercise programme was considered to be at 16 weeks or 48 sessions. Attendance was logged by project staff.
Tiedmann, Sherrington & Lord, 2011. Australia [37].	Examine whether a diverse array of physiological, psychological, health and lifestyle measures are associated with exercise adherence in older retirement	Exploratory Intervention Study (pre/post)	N=344 (aged 62 and over) No other details available.	**Intervention:** group exercise or yoga classes twice a week for 6–12 months.	**Measure:** low adherers (those who attended less than 30% of exercise classes.

	village residents				
Shubert et al, 2011. U.S [41].	To translate a research-based intervention into a community programme, and to assess if similar outcomes were achieved.	Exploratory Intervention Study (pre/post)	N=68 (mean age= 78.8) 77.5% women 74% white Caucasian (non-Hispanic), community dwelling.	**Intervention** Stay Safe, Stay Active" (SASA). 60-75 minute, 12 week strength and balance class (2 classes offered twice a week).	**Measure:** Completion of the programme was defined as attending 75% (18) or more classes
Seymour et al, 2009. U.S [26].	To test the impact of a shift in instruction type (from physiotherapist to exercise instructor) on participant outcomes	Exploratory Intervention/2 group pre/post-test design study	N=161, PT group N=190, CEI (mean age= 71.2) 53.2% African American. healthy community dwelling	**Intervention:** 2 identical multi-component exercise classes, 90 minutes, 3 times a week, 8 weeks. Intervention delivered by: Therapist (PT) or exercise instructor (CEI).	**Measure:** based on attendance of 8 week intervention documented at sessions.<br><br>Also, Community Healthy Activities Model Program (CHAMPS) measure to assess maintenance of physical activity (self-report) after intervention.
Hawley-Hague et al, 2014 U.K. [10]	To examine the influence of individual, instructor and group factors on participants' attendance and adherence to community exercise classes for older adults.	Longitudinal cohort study	N=193 (mean age= 76.1), 90.7% women, 94.3% white British. Community dwelling.	**Intervention:** Recruited existing multi-component community exercise classes. Including some community falls prevention. Delivered once a week for 60 minutes plus social time afterwards.	**Measure:** *Attendance in weeks.* Weekly class attendance records provided by the instructor.<br>*Adherence.* Adherence levels calculated at each follow-up period. Non-adherence was defined as "those not attending at follow-up and have not attended for 4 weeks, and have not given a reason for nonattendance or those who

					have stated they are dropping out.”
Hicks et al, 2012. Italy [40].	To identify factors that were predictive of improved pain status among older adults with chronic back pain participating in the Adaptive Physical Activity (APA) program and to identify factors that were predictive of adherence to APA	Observational Cohort study	N=392 (mean age=66.8) 84.2% women. No ethnicity stated. Community dwelling.	**Intervention**<br><br>The APA multi-component exercise programmes were held twice a week for 1 hour.	**Measure:** Defined as participation in >75% of all exercise sessions for the entire12-month study period
Lucidi et al, 2006. Italy [35]	Whether, and to what extent, the constructs implicated in the theory of planned behaviour could predict behavioural intention to exercise and exercise-class attendance of older adults (age 65–90 years) already enrolled in a physical activity programme	Cross sectional study	N = 1,095 (mean age=69) Community dwelling	2 multi-component classes a week. Participants enrolled in exercise class for minimum of 6 months. Intervention delivered by: physical trainers.	**Measure:** Percentage ratio obtained by dividing the number of attended sessions by the number of possible sessions over the 3-month period. Registers used.
Sin et al,	Evaluate feasibility	Exploratory	N= 13, (mean	Evidence based exercise	**Measure:** Adherence to the

2005. U.S [25]	and effectiveness of a modified exercise programme for elderly Korean immigrants	intervention study	age=77) 100% Korean immigrants supported housing.	class 3 times a week, 12 weeks. Intervention delivered by: Korean/American exercise instructor.	exercise programme was calculated as percent of exercise classes attended over 12 weeks. Registers used.
Hays et al, 2005. U.S [33].	Explore the relationships between exercise self-definitions and participation in a group-based exercise programme	Exploratory intervention study	N=192 (mean age=64) 64.2 African-American 92% hypertension community dwelling	Multi-component community class available 2 times a day, 5 days week.	**Measure:** Mean number of exercise sessions attended over 24 weeks. A *completed session* was defined as a minimum of 20 minutes of continuous exercise at 55% to 70% of maximal heart rate (moderate intensity as defined by the ACSM,^' 1995) as determined by the research assistant who was present at each exercise session.
Hughes, Seymour et al, 2004. U.S [50]	Assess impact of low cost, multi-component physical activity intervention.	Randomised Controlled Trial	N=80 (mean age=73.5). 81.0% women 84.6% white Caucasian mild/moderate osteoarthritis. community dwelling	**Intervention:** multi-component class and behaviour change, 90 minutes, 3 times a week, 8 weeks. Intervention delivered by: Physical Therapists. **Control:** Waiting list.	**Measure:** Monitored attendance at sessions but they were asked to log activity. Frequency (number of times a week) and duration (minutes). Analysis was mostly done with self-report data.
Tu et al, 2004. U.S [18].	Investigates the effects of health and environmental factors on the dropout and intermittent	Exploratory intervention study	N=110 (mean age=63.7) 100% women. 66% African American, 34% white	2 multi-component classes each day offered 5 days a week for 2 years.	**Measures:** Number of days between the patient's first and last attended exercise classes (i.e., time until dropout) or the date of the last class offered

	nonattendance of an exercise programme designed specifically for older, female, primary-care patients living in the inner city		Caucasian. community dwelling		Participant's daily attendance record (1 = attended, 0 = did not attend) starts with first attendance and concludes with last attended class.
Litt et al, 2002. U.S [52]	To determine the extent to which modifiable social learning constructs predicted long-term adherence to an exercise programme.	Exploratory intervention study	N=189 (mean age=67.4) randomised to upper (n=92) or lower body (n=97) exercise. 100% women no ethnicity stated low bone density. community dwelling	2 classes a week for 2 months, 1 a week for 2 months, 1 a fortnight for 2 months. Then two classes a month.	**Measure:** Participants were asked the number of days in the previous 30 on which they had exercised, at moderate intensity, as per the prescribed regimen. Verified by examination of bi-weekly exercise logs. Analysis looked at "days of exercise".
Nigg et al, 2002. U.S [27]	To investigate effects of a community-based physical activity intervention grounded in the Transtheoretical Model	Exploratory intervention study	N= 48, (mean age 78.2) 90% women, 98% white Caucasian community dwelling	Pamphlets on benefits of exercise and 45 min in-house exercise class 2 times a week for 7 months.	**Measure:** Regular attendance, attending a minimum of 80% of the sessions.<br><br>Based on movement between stages of change (TTM). Participants were labelled improved, unchanged, declined.
Estabrooks et al, 1999. Canada [16]	Examined the relationship of group cohesion to attitude and control	Exploratory intervention study	N=179 (mean age=67) 73% women no ethnicity	Existing classes 2-3 times a week, mix of strength, cardiovascular, walking and tai chi.	**Measure:** Attendance was converted to a percentage of total classes available over the 4-week period

	beliefs toward exercise in a sample of older adults		stated healthy community dwelling	Followed for 4 weeks.	
Estabrooks et al, 1999 Study 2. U.S [15]	Examine the effectiveness of team building intervention for improving exercise class attendance of previously sedentary older adults.	Exploratory intervention study	N= 33 (mean age= 75.1) N=12 intervention, placebo N=11, control N=11. 91% women no ethnicity stated healthy sedentary community dwelling.	Exercise class including instructor led team building, 2 times a week, 6 weeks. **Placebo**: Exercise class 2 times a week, 6 weeks with visit from researcher enquiring how participants progressing. **Control:** Basic exercise class 2 times a week, 6 weeks.	**Measure:** Adherence based on attendance over 6 weeks (percentage of classes attended) but also return rate after 10 week break.
Grove and Spier, 1999. U.S [36]	To evaluate the usefulness of intervention strategies in motivating adherence to an exercise programme.	Exploratory intervention study	N=14 (mean age= 78) 100% women 100% white Caucasian healthy community dwelling	Multi-component exercise group 2 times a week. Delivered by: exercise video with support from a nurse for 6 weeks, then once a week for 4 ½ months and a peer captain after 6 weeks.	**Measure:** Percentage of older adults who attended more than 50% and 90%-100% of sessions
Brenes & Storandt, 1998. U.S.[47]	To examine the effectiveness of the theory of planned behaviour in predicting exercise by older adults 1,3, and 9 months after beginning an	Exploratory intervention study	N= 105 (mean age= 68.3) 89% women. 66% white Caucasian 31% African-American healthy	Older adults new to exercise attending existing exercise groups. Not stated how often classes offered.	**Measure: Self report.** Participants rated on a 7-point scale ***(disagree*** to ***agree)*** the following statement: "I have exercised 2 to 3 times a week over the past 1 (3, 9) month(s)."

	exercise class.		community dwelling		
Caserta & Gillett, 1998 U.S [48]	Determine acute and follow-up effects of 4 months of health and fitness education with and without exercise.	Randomised Controlled Trial	N=76 exercise group (mean age=64.7). 100% women 98% white Caucasian sedentary, overweight community dwelling.	**Intervention:** exercise/education programme 3 times a week, 16 weeks Intervention delivered by: Nurse. **Control:** Health education sessions were encouraged to exercise 3 times a week at home, 16 weeks.	**Measure:** Self-report diaries. 3 times a week for 30 minutes at 60-80% of MHRR (although this is not clear in the paper).
Ecclestone et al, 1998. Canada [21]	Examine the pattern of enrolment, attendance and adherence across a variety of programmes.	Exploratory intervention study	N=67 and N=31 (mean age=75 and 73) 96.5% women no ethnicity stated healthy community dwelling.	2 Osteoporosis classes offered 3 times a week for 3 years. Intervention delivered by: instructors majority of which are peer (55+) leaders.	**Measure:** Percentage of classes attended divided by the actual number of sessions offered for each class in each calendar month.<br>**Drop out defined as:** Not registered on any programmes, not attending a single session over a 12 month period and not returning to a session over the 12 month tracking period.
Mills et al, 1997. U.S [28]	Explores activity preference in relation to activity adoption and maintenance.	Exploratory intervention study	N=98 (mean age=76) 81% women, 92% white Caucasian fairly healthy community dwelling	CHAMPS programme, intervention promoting exercise including conditioning classes, provided counselling, follow-up calls, monthly group meetings, for 6 months	**Measure:** Self-report activity logs, a sample of the most popular class registers were used to validate attendance. Maintained participation, described as attending at least one class a month, assessed through self-report, but validated by

			sheltered housing.		attendance records
Williams and Lord, 1995. Australia. [20]	Whether psychological, physiological, and health and lifestyle measures were associated with adherence to a structured exercise programme for older women	Exploratory intervention study	N=102 (mean age=71.6) 100% women no ethnicity stated healthy community dwelling	5 multi-component classes, 1 hour twice a week for 12 month trial.	**Measure:** Adherence was defined as the number of exercise classes attended.<br><br>**Dropout:** used to describe participants who withdrew from the formal exercise programme.
Hickey et al, 1995. U.S [32]	Effectiveness of low-intensity physical activity for improving functional ability and psychological well-being in chronically impaired older individuals<br><br>Exploring initiating and maintaining an exercise programme for physically inactive persons who are limited by chronic impairments	Exploratory intervention study	N=90 (mean age=72.6) 94% female 52% white Caucasian 48% black chronically impaired, sedentary, senior centres	4 structured low-intensity exercise classes, 2 times a week by instructor for 6 weeks, followed by peer led group with instructor support up to 18 weeks.	**Measure:** Adherence if they attended two thirds of the sessions.
Howze et al, 1989. U.S [34]	Examines factors affecting the adoption of regular	Exploratory multiple intervention	N=47 (mean age= 65.3), mixed gender,	Multi-component exercise programme. Running for 1 month	**Measures:** Number of attendances. High attenders categorised as 15/20 sessions, low

	exercise by sedentary older adults	percentage not stated. 100% white Caucasian healthy community, middle class.	before data collection (no further detail about classes).	attenders were less than 15/20.
